# The Effectiveness of Ultrasound-Guided Subacromial-Subdeltoid Bursa Combined With Long Head of the Biceps Tendon Sheath Corticosteroid Injection for Hemiplegic Shoulder Pain: A Randomized Controlled Trial

**DOI:** 10.3389/fneur.2022.899037

**Published:** 2022-06-14

**Authors:** Yajing Hou, Tong Zhang, Wei Liu, Minjie Lu, Yong Wang

**Affiliations:** ^1^Rehabilitation Medicine Center, Fuxing Hospital, Capital Medical University, Beijing, China; ^2^School of Rehabilitation Medicine, Capital Medical University, Beijing, China; ^3^Department of Neurological Rehabilitation, Beijing Bo'ai Hospital, China Rehabilitation Research Center, Beijing, China

**Keywords:** hemiplegic shoulder pain, subacromial-subdeltoid bursa corticosteroid injection, biceps tendon sheath corticosteroid injection, combined therapy, ultrasound-guided

## Abstract

**Background:**

Subacromial-subdeltoid (SASD) bursa and long head of the biceps tendon (LHBT) sheath corticosteroid injection are commonly used to treat shoulder pain associated with arthritic shoulder conditions, but effectiveness in the stroke population is unclear. This study aimed to investigate the clinical effectiveness of ultrasound-guided SASD bursa combined with LHBT sheath corticosteroid injection for hemiplegic shoulder pain (HSP) compared with SASD bursa injection alone.

**Methods:**

60 patients with HSP were randomly allocated to the dual-target group (*n* = 30) and single-target group (*n* = 30). The single-target group received SASD bursa corticosteroid injection alone, and the dual-target group received SASD bursa and LHBT sheath corticosteroid injection. The primary endpoint was pain intensity measured on a visual analog scale (VAS). The secondary endpoint was passive range of motion (PROM) of the shoulder, Upper Extremity Fugl-Meyer assessment (UEFMA) score, and Modified Barthel Index (MBI) score. PROM and pain intensity VAS were assessed at baseline and weeks 1, 4, and 12 post-treatment. UEFMA and MBI were recorded at baseline and weeks 4 and 12 post-treatment.

**Results:**

A total of 141 patients with HSP were screened, and 60 patients were included. Significant differences in the VAS, PROM, UEFMA and MBI were observed at all follow-ups in both groups. The dual-target group showed a significant difference in VAS score compared with the single-target group (3.3 vs. 3.7, *p* = 0.01) at week 4 and week 12 (2.5 vs. 3.2, *p* < 0.001). Moreover, the dual-target group showed statistically significant differences in flexion (*p* < 0.001) at week 12, extension rotation (*p* < 0.001) at week 12, and abduction at week 1 (*p* = 0.003) and weeks 4 and 12 (*p* < 0.001) compared with the single-target group. There were significant differences in FMA and MBI scores in the two groups before and after treatment (*p* < 0.001), with a more significant increase in the dual-target group compared with the single-target group (*p* < 0.001) at week 12.

**Conclusion:**

The combination of SASD bursa and LHBT sheath corticosteroid injection is superior to SASD bursa injection alone in reducing shoulder pain and improving functional activities in patients with HSP.

**Clinical Trial Registration:**

www.chictr.org.cn, Unique identifier: ChiCTR2100047125.

## Introduction

Hemiplegic shoulder pain (HSP) (1–4) which is a common complication after stroke may affect the rehabilitation process and decrease activities of daily living (ADL). Evidence from a systematic review suggests that the prevalence of HSP varies from 22 (2, 5) to 47% (6). It has been reported that HSP can develop as early as 2 weeks poststroke but typically occurs 2–4 months post-stroke. HSP (7) leads to shoulder pain and restriction of motion (8), which is the main reason for poor recovery of arm function (9), depression (6, 10), and disturbed sleep. Moreover, shoulder pain has also been associated with prolonged hospital stays, higher healthcare bills (11, 12), and persistent disability and ~29% of patients continue to have pain or functional limitations for up to 1 year. The main aims of treatment are to reduce pain, restore shoulder mobility, improve functional activities, and prevent progressive degenerative changes. However, although more and more treatment methods are widely used in the treatment of HSP (13), such as Botulinum toxin type A (14, 15), electrical stimulation (16, 17), and suprascapular nerve block (SSNB) (18, 19), there is no consensus about which treatment method is the best way to manage HSP. As identifying the exact etiology of shoulder pain can be difficult, the management of HSP is challenging.

The causes of HSP are multifactorial. In addition to loss of motor control, central sensitization (20) and spasticity (14), soft tissue lesions such as rotator cuff tears, bicipital tendinitis, subacromial subdeltoid (SASD) bursitis, and muscle imbalance may also play a key role (21). SASD bursitis, which is important among the factors involved in the occurrence of HSP (21, 22), is the most common cause of pain and functional disability in this population. It is known to all that SASD bursa corticosteroid injection is commonly used in the management of shoulder pain (23). In fact, corticosteroid injection has been demonstrated to be an effective method for HSP and provides a faster improvement in shoulder PROM by reducing local tissue inflammation and pain (24–26). In a randomized, triple-blind, placebo-controlled trial, which investigated the efficacy of subacromial corticosteroid injection on poststroke shoulder pain, significant improvements in pain, disability, and active range of motion lasting as long as 8 weeks were observed in patients treated with corticosteroid injection, but not in those who received injection with lidocaine only (24). However, there is no significant difference in activities of daily living between the two groups in this study. Some researchers believe that injection technology and complex causes of shoulder pain after stroke are the main reasons for limited functional improvement which gains from an SASD corticosteroid injection alone.

An important–but easily overlooked–structure is the long head of the biceps tendon (LHBT), which has been reported as an important pain generator and a common location of anterior shoulder pain (27–30). Previous studies have shown that biceps tendon disorders are associated with rotator cuff tears, subacromial bursitis, and dynamic shoulder instability. Stroke is a common neurologic disorder causing severe paralysis, which can alter the normal protective movement patterns of the shoulder joints and also lead to severe muscle weakness and rotator cuff tears. In patients with HSP, LHBT tendinitis or effusion (31) are the most common causes of shoulder pain, accounting for 49% of cases (32). Moreover, according to MRI results, SASD bursitis and bicipital tendinitis may coexist in patients with HSP. Several studies showed that corticosteroid injection into the LHBT sheath was a safe procedure that reduced pain and improved shoulder function in patients with LHBT tendinitis (33–35).

Although studies have shown that corticosteroid injections into SASD bursa and the LHBT sheath are safe and useful for non-stroke patients with shoulder pain (36), the combination of the SASD bursa and LHBT sheath corticosteroid injection in the treatment of HSP has not yet been investigated. It would be reasonable to evaluate the efficacy of a combination of different treatment methods to result in improvement of their effects in the management of HSP. Therefore, the assumption is that the combination of SASD bursa and LHBT sheath corticosteroid injection might provide better results in pain and shoulder function when compared with SASD bursa alone. The main purpose of this study was to explore the clinical effectiveness of a combination of SASD bursa and LHBT sheath corticosteroid injection for HSP.

## Methods

The study design was a parallel-group, randomized controlled trial. A total of 60 participants signed informed consent before participation and were randomized to the dual-target group (*n* = 30) or the single-target group (*n* = 30). All patients in both groups received physical therapy for shoulder and upper limb rehabilitation training. The trial was registered in the Chinese Clinical Trial Registry with the registration number ChiCTR2100047125.

### Setting

All methods were carried out following the approved ethical guidelines. The study protocol was approved by Fu Xing Hospital Ethics Committee (Approval Notice Number 2021FXHEC-KY032).

### Participants and Eligibility Criteria

#### Inclusion Criteria

(1) Post-stroke duration ≥ 1-month, but < 12-months; (2) aged 18 to 75 years; (3) ultrasonographic findings: subacromial bursa thickness >2 mm and/or effusion thickness >2 mm (37) and LHBT thickening, tenosynovitis/hypertrophy of the synovial sheath, and fluid surrounding the tendon in the groove (38, 39); (4) Neer test and bicipital groove compression test (+); (5) score on the visual analog scale (VAS) of shoulder pain ≥3; (6) a minimum score of 20 points for the Mini-Mental State Examination to ensure that patients could make their own decisions to participate in the research and report changes in pain; (7) patients received SASD bursa and/or LHBT sheath corticosteroid injection.

#### Exclusion Criteria

(1) Severe aphasia or cognitive impairment precluding accurate clinical assessment of VAS score; (2) severe spasticity using the Modified Ashworth Scale (grades 3 and 4); (3) patients had received corticosteroid injection for shoulder pain in the past month; (4) hypersensitivity to injection agents; (5) hemiplegic shoulder pain caused by thalamic damage; (6) a modified Ashworth scale (MAS) score of 3 or more points for spasticity in subscapularis muscle, biceps brachii, pectoralis major, and pectoralis minor; and (7) The presence of another obvious explanation for the pain (fracture, ligament injury, and adhesive capsulitis).

### Randomization, Treatment Allocation, and Blinding

We have estimated that our sample size was 60, and we set 15 blocks with 4 patients in a block. For every block, the randomization number was allocated to 2 bigger numbers and 2 smaller numbers, and patients allocated to the smaller number would receive SASD bursa and LHBT sheath injection, otherwise, they would receive SASD bursa injection alone. The randomization number was generated using a computer, and the investigator who assigned the randomization number is blinded to the study, he only knew the allocation number and told the physicians whether the number was bigger or smaller. Considering that LHBT sheath physiological saline injection is invasive, the single-target group who is allocated to receive SASD bursa injection alone did not receive LHBT sheath physiological saline injection in order to meet the ethical requirements. As a result, the physicians and patients were not blinded to the study, and outcome assessors and trial statisticians were blinded to the treatment for the duration of the study.

### Intervention

When patients were eligible for inclusion, baseline characteristics would be collected. Three days after hospitalization, eligible patients received treatment according to the randomization consequence. Under ultrasound guidance, all injection treatments were performed by the same operator who is an experienced and board-certified physician. Both groups received routine rehabilitation treatment. After treatment, all patients received a standard course of exercise therapy (capsular stretching, ability of daily living exercise, increasing active and passive motion, and so on) and physiotherapy (neuromuscular electrical stimulation and ultrashort wave therapy), 24 h after injection during the 4 weeks. During this period, participants were asked to do their exercises 5 days per week (20–30 min per time) by a physical therapist blinded to the study.

#### LHBT Sheath Injection

**Figure 1:**
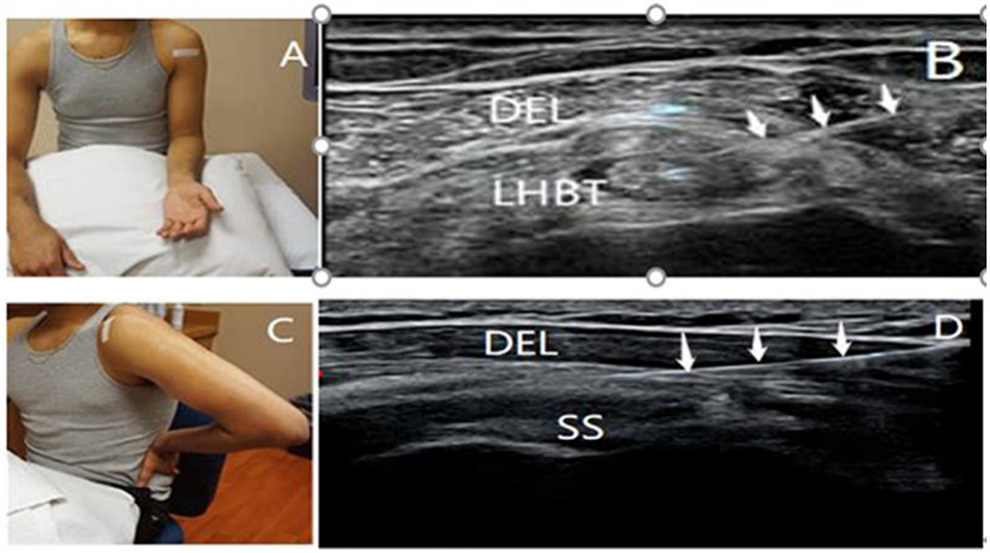
Patient's position and ultrasonography image of SASD bursa and LHBT sheath injection. The patient was positioned to obtain views of the long head of the biceps tendon **(A)** and subacromial-subdeltoid bursa **(C)** during the ultrasound-guided injection. The white tape indicates the position of the transducer. Ultrasound images showing the needles inserted into the proximal bicipital tendon sheath **(B)** and subacromial-subdeltoid bursa **(D)** (↓, needle; DEL, deltoid; SASD, subacromial-subdeltoid; SS, supraspinatus tendon; LHBT, the long head of the biceps tendon).

Injection drugs were prepared in advance: 1 ml of compound betamethasone injection (CBI) + 1 ml of 2% lignocaine injection. During the treatment, patients sat upright with their forearm in a supination position and shoulder in neutral position (Figure 1A). The upper limb of the affected side was kept with the palm facing upwards and close to the side of the body, and the forearm was kept bent at 90° after rotation. The affected shoulder joint was completely exposed, and a transducer was placed along the short axis of the LHBT between the lesser and greater tuberosities. After disinfection and cleaning, the physician selected a specific puncture needle and syringe (5 ml) and inserted the needle from the lateral to the medial direction to reach the biceps tendon sheath by piercing the coracohumeral ligament (Figure 1B). For patients with tendon sheath effusion, the effusion was drained first, and then 2 ml of the prepared medicine was carefully injected into the LHBT sheath once no blood was detected.

#### SASD Bursa Injection

Injection drugs were prepared in advance: 1 ml of compound betamethasone injection (CBI) + 2 ml of 2% lignocaine injection + 1 ml of normal saline. During the treatment, patients' hands were positioned on the buttock to induce internal rotation and hyperextension of the shoulder, and the elbow was bent (40) (modified Crass position) (Figure 1C). The affected shoulder joint was completely exposed, and a transducer was placed along the long axis of the supraspinatus tendon beneath the acromion in the modified Crass position. The physician selected a puncture needle and inserted it using a lateral to medial approach, following disinfection and cleaning. With the aid of high-resolution ultrasound, the needle was introduced to reach the SASD bursa located between the deltoid muscle and supraspinatus tendon (41) (Figure 1D). For patients with SASD bursa effusion, the effusion was drained, and 4 ml of the prepared medicine was accurately injected into the SASD bursa once no blood was detected. The needle was withdrawn immediately after the injection, and a sterile dressing was applied at the injection point. The site was kept dry for 24 h after injection. Patients who experienced discomfort after injection were treated with local cold therapy, and patients without discomfort had their dressing removed after 24 h.

#### Outcomes

Participants were assessed at baseline and then in weeks 1, 4, and 12 after injection. Demographic data were collected, including age, sex, etiology, onset date of stroke, and lesion site. The primary endpoint was pain relief, measured by VAS. A previous study has shown that patients require a 1.4-cm reduction in the VAS pain score to achieve a minimal clinically important difference (42). In addition, the treatment was considered to have yielded good results when the patient's VAS score was <3.33 cm (i.e., mild pain) or when the score was less than half of the initial score (43). The secondary endpoints were disability and ADL, measured by the pain-free passive range of motion (PROM) of the shoulder, the upper extremity Fugl-Meyer Assessment (FMA,) and Modified Barthel Index (MBI). The pain-free PROM of the affected shoulder was measured using handheld goniometry. These measurements included abduction in the frontal plane, forward flexion, internal rotation, and external rotation. Measurement sensitivity was set at 5°. The recovery of motor function was assessed using FMA (32 items, 0–2 points for each item; score range: 0–66), where higher scores indicate better function. ADL was evaluated using the MBI, composed of 10 items (personal hygiene, bathing, feeding, toileting, up/down stairs, dressing, defecation, voiding, ambulation, and chair/bed transfer) on a scale of 0 to 100, with higher scores indicating higher levels of independence. PROM and pain intensity were evaluated before treatment and at weeks 1, 4, and 12 of follow-up visits. FMA and MBI were recorded at baseline, week 4 and week 12 post.

#### Sample Size and Statistical Analysis

This research is powered for the primary outcome measure of VAS score. We used PASS 15 software to calculate the sample size, which was based on a statistically and clinically significant difference in the VAS score at 1 week between the two groups. Based on the results of previous studies, we assumed that the VAS score would be 4.5 ± 1.8 in the single-target group and 3.1 ± 1.7 in the dual-target group at 1 week after injection. There would be a difference between groups of 1.4 points in the mean VAS pain score at 1 week two-sided-side 0.05 level of significance and a sample size of 56 patients (28 per in each group) provided 80% statistical power or demonstrate this difference in VAS score. Considering a dropout rate of 15%, we will recruit a total of 60 participants (30 per group).

All the statistical studies were performed based on the principle of intention-to-treat analysis. This study summarized continuous variables as Mean ± SD, and categorical data were presented as numbers and percentages. Shapiro-Wilk tests were performed to determine the normality of the data distribution. Independent *t*-tests (in case of normal distribution) or Mann–Whitney U tests (in case of non-normal distribution) were used to compare the average value of the continuous variables at baseline between the two groups. The categorical data at baseline were compared using the chi-squared test or Fisher exact. The intragroup comparisons in continuous variables during the pretreatment (baseline) and post-treatment (week 1, week 4, and week 12) periods were analyzed with the related samples Wilcoxon signed-rank test, and intergroup comparisons were performed with the independent-samples Mann–Whitney U test. A linear mixed model was used to analyze any significant interactions between pain scores and UEFMA (or MBI) scores in the two groups. In order to control type-1 error, we performed Bonferroni correction in all possible multiple comparisons and the results were considered statistically significant at a level of *p* < 0.016 in intragroup comparisons. The level of significance was set as a 2-sided *p* < 0.05. All analyses were conducted with SPSS version 25.0.

## Results

**Figure 2:**
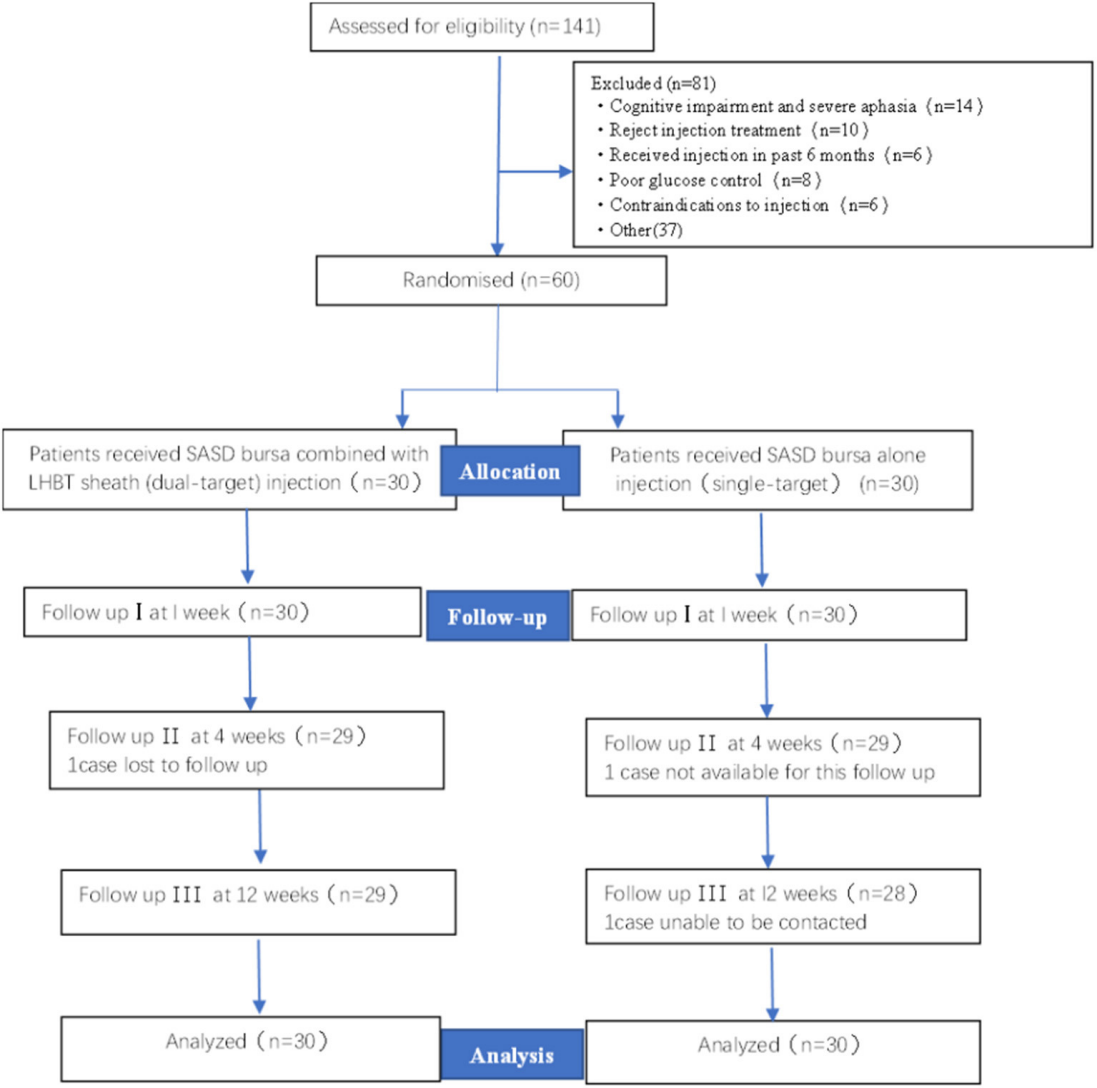
Flow chart of study enrollment. SASD, subacromial-subdeltoid; LHBT, the long head of the bicep tendon.

A total of 141 patients participated after giving informed consent and undergoing screening for HSP. All 60 eligible participants were randomized to the single-target (*n* = 30) or dual-target (*n* = 30) groups and included in the data analysis. One participant from the single-target group and one participant from the dual-target group missed the follow-up visit at week 4 and week 12. One participant in the single-target group lost contact with the study organizers at the 12-week visit. The flowchart summary of the study is shown in Figure 2. No side effects were reported in this study. The baseline demographic data of the patients are summarized in Table 1. There were no significant differences between the two groups in demographics, stroke type, and time since stroke onset (*p* > 0.05).

**Table 1:** Baseline characteristics between the two groups.

**Characteristics**	**Dual-target injection group (*N* = 30)**	**Single-target injection group (*N* = 30)**	**P**
Age (y) (mean ± SD)	55.2 ± 10.2	56.8 ± 13.1	0.276
Duration of stroke (m) (mean ± SD)	3.1 ± 1.9	2.6 ± 1.4	0.141
Sex (*n*, %)			0.321
Male	17 (56.7)	16 (53.3)	
Female	13 (43.3)	14 (46.7)	
Etiology (*n*, %)			0.400
Hemorrhagic	13 (43.3)	14 (46.7)	
Ischemic	10 (33.3)	13 (43.3)	
Others	7 (23.3)	7 (23.3)	
Lesion site (*n*, %)			0.918
Left	14 (46.7)	13 (43.3)	
Right	16 (53.3)	17 (56.7)	
Site of injury (*n*, %)			0.726
Basal ganglia	14 (46.7)	14 (46.7)	
Frontal and temporal lobe	7 (23.3)	11 (36.7)	
Brainstem	5 (16.7)	3 (10.0)	
Epencephalon	2 (6.7)	1 (3.3)	
Subarachnoid hemorrhage	2 (6.7)	1 (3.3)	

*Others: glioma, subarachnoid hemorrhage, https://www.geenmedical.com/article?id=33625724&type=true*.

### Primary Outcomes

Our primary and secondary results are shown in Table 2. There was no significant difference in baseline VAS scores between the two groups (*p* = 0.546). In comparison with the baseline, a significant decrease in VAS scores was observed in the within-group comparisons of both groups (*p* < 0.001 at week 1; *p* < 0.001 at week 4; *p* < 0.001 at week 12, for both groups). There were no significant changes in VAS scores at week 1 between the two groups (4.4 vs. 4.5, *p* = 0.475). However, the dual-target group showed a statistically significant difference in VAS score compared with the single-target group (3.3 vs. 3.7, *p* = 0.01) at week 4, and week 12 (2.5 vs. 3.2, *p* < 0.001). In addition, at week 4-FU, the VAS in the dual-target group decreased to 3.3 at week 4-FU and achieved a standard of good results, but the VAS in the single-target group didn't meet the standard. What's more, at week 4-FU, the VAS improvement in the dual-target group is higher than in the single-target group, the relief degree is 64 vs. 52%.

**Table 2:** Comparisons all the measured variable between the groups.

**Measured variable**	**Dual-target injection group (*****N*** **=** **30)**		**Single-target injection group (*****N*** **=** **30)**		**Between 2 groups**
	**Mean ± SD**	***P* value**	**Mean ± SD**	***P* value**	***P* value**
**VAS baseline**	6.9 ± 1.2		6.7 ± 1.0		0.546
Week 1-FU	4.4 ± 1.0	<0.001*	4.5 ± 0.9	0.001*	0.475
Week 4-FU	3.3 ± 0.9	<0.001^†^	3.7 ± 0.7	0.001^†^	0.010
Week 12-FU	2.5 ± 0.9	<0.001^‡^	3.2 ± 0.8	0.001^‡^	<0.001
**Passive FL baseline**	97.6 ± 15.4		101.2 ± 15.7		0.368
Week 1-FU	115.6 ± 12.5	<0.001*	114.5 ± 14.0	<0.001*	0.468
Week 4-FU	125.6 ± 11.2	<0.001^†^	122.7 ± 13.2	<0.001^†^	0.139
Week 12-FU	141.3 ± 7.8	<0.001^‡^	133.1 ± 11.5	<0.001^‡^	<0.001
**Passive Abd baseline**	81.7 ± 12.6		81.9 ± 13.0		0.862
Week 1-FU	96.8 ± 13.0	<0.001*	90.1 ± 13.0	<0.001*	0.003
Week 4-FU	107.1 ± 12.0	<0.001^†^	96.5 ± 13.0	<0.001^†^	<0.001
Week 12-FU	122.0 ± 12.0	<0.001^‡^	105.0 ± 13.0	<0.001^‡^	<0.001
**Passive EX baseline**	34.2 ± 8.9		34.7 ± 8.9		0.976
Week 1-FU	42.6 ± 8.1	<0.001*	41.4 ± 8.6	<0.001*	0.352
Week 4-FU	49.1 ± 7.1	<0.001^†^	47.0 ± 7.8	<0.001^†^	0.096
Week 12-FU	59.1 ± 6.6	<0.001^‡^	53.4 ± 5.8	<0.001^‡^	<0.001
**Passive IN baseline**	47.4 ± 8.3		46.6 ± 8.3		0.434
Week 1-FU	56.8 ± 6.1	<0.001*	54.0 ± 7.4	<0.001*	0.085
Week 4-FU	62.0 ± 4.9	<0.001^†^	60.0 ± 6.3	<0.001^†^	0.098
Week 12-FU	67.0 ± 3.2	<0.001^‡^	65.0 ± 5.0	<0.001^‡^	0.074
**FMA score baseline**	17.1 ± 3.9		16.0 ± 3.4		0.131
Week 4-FU	24.9 ± 4.6	<0.001^†^	22.7 ± 3.4	<0.001^†^	0.0009
Week 12-FU	35.7 ± 4.3	<0.001^‡^	31.5 ± 4.4	<0.001^‡^	<0.001
**MBI score baseline**	33.7 ± 8.5		34.0 ± 8.0		0.768
Week 4-FU	50.9 ± 8.4^†^	<0.001^†^	47.9 ± 7.5	<0.001^†^	0.099
Week 12-FU	66.1 ± 7.9	<0.001^‡^	62.1 ± 6.8	<0.001^‡^	0.010

*Values represent the mean ± standard deviation. VAS, visual analog scale; FU, follow up; FL, flexion; Abd, abduction; EX, extension; IN, internal rotation; UEFMA, Upper Extremity Fugl-Meyer Assessment; MBI, Modified Barthel. P values pertain to between-group comparison for difference from baseline.*

* *Significant difference between baseline and one week posttreatment in the same group (p <0.05).*

† *Significant difference between baseline and weeks 4 posttreatment in the same group (p < 0.05).*

‡ *Significant difference between baseline and weeks 12 posttreatment in the same group (p < 0.05)*.

### Secondary Outcomes

There were no significant differences in baseline shoulder PROM, FMA, and MBI scores between the two groups (*p* > 0.05). In comparison with baseline results, significant differences in the shoulder PROM, FMA, and MBI scores were observed in the within-group comparisons in both groups (*p* < 0.001 at week 1; *p* < 0.001 at week 4; *p* < 0.001 at week 12, for both groups). The intergroup comparison revealed that increases in some shoulder PROMs were significantly higher in the dual-target group compared with the single-target group. There were significant differences in the change in shoulder PROM measurements of maximum flexion angle at week 12, (141.3 ± 7.8 vs. 133.1 ± 11.5, *p* <.001), maximum abduction angle at week 1, week 4, and week 12. (96.8 ± 13.0 vs. 90.1 ± 13.0, *p* = 0.003; 107.1 ± 12.0 vs. 96.5 ± 13.0, *p* < 0.0001; 122.0 ± 12.0 vs. 105.0 ± 13.0, *p* < 0.001), and maximum extension rotation angle at week 12 (59.1 ± 6.6 vs. 53.4 ± 5.8, *p* < 0.001).

Intergroup comparisons revealed that the increase in the FMA score was statistically significant at week 4 (*p* = 0.009) and week 12 (*p* < 0.001), with the dual-target group showing higher scores than the single-target group (Table 2). Although the MBI score was greater in the dual-target group than in the single-target group at week 4, the differences among groups did not reach statistically significant levels (*p* = 0.099) (Table 2). Only the dual-target group showed a significantly higher MBI score at week 12 (*p* = 0.01) than the single-target group (Table 2).

As summarized in Table 3, VAS scores of the dual-target group were negatively correlated with FMA score (β = −2.138 < 0; *p* < 0.001) and MBI score (β = −5.165 < 0; *p* < 0.001). This was also true for the single-target group for FMA score (β = − 2.340, *p* < 0.001) and MBI score (β = −5.199; *p* < 0.001). Compared with the single-target group, the association of pain intensity with FMA score (β = 0.202) and MBI score (β = 0.034) was more significant in the dual-target group (*p* < 0.001).

**Table 3:** Linear mixed model analysis interaction of group *VAS score.

**Dependent variable**	**Dual-target injection group (*****N*** **=** **30)**		**Single-target injection group (*****N*** **=** **30)**		**Dual-target injection-single-target injection**	
	**β**	***P***	**β**	***P***	**β**	***P***
FMA score	−2.138	<0.001	−2.340	<0.001	0.202	<0.001
MBI score	−5.165	<0.001	−5.199	<0.001	0.034	<0.001

* *Independent variable, VAS score; VAS, visual analog scale; MBI, modified Barthel index; UEFMA, Upper Extremity Fugl-Meyer Assessment*.

## Discussion

In this prospective study, we hypothesized that SASD bursa in combination with LHBT sheath corticosteroid injections might provide a better outcome in the patients with HSP. According to the present research results, significant improvements in pain, PROM, UEFMA, and MBI were observed in both groups after treatment. However, compared with the single-target injection, VAS, flexion, external rotation, UEFMA, and MBI score showed significant improvements in the dual-target injection group. Therefore, the main finding of this study showed that SASD bursa plus LHBT sheath corticosteroid injections were more effective in reducing pain and improving functional activities than SASD bursa alone. In addition, pain relief seems to have a positive effect on shoulder PROM, the upper limb function, and activities of daily living (ADL). To our knowledge, this is the first study to evaluate the effectiveness of ultrasound-guided SASD bursa combined with LHBT sheath corticosteroid injection for reducing pain, and improving PROM in patients with HSP.

The SASD bursa is essential for shoulder movement and plays a key role in the subacromial gliding mechanism, which is covered by suprascapular nerve and free nerve endings (44). Several previous studies have shown that proinflammatory cytokines (IL-1β and IL-6), metalloproteases (MMPs) (45), and pain mediators (COX-2 and substance P) were associated with shoulder pain in patients with SASD bursitis. It has been demonstrated in several studies that SASD bursitis, an inflammation of the SASD bursa, is one of the most common causes of poststroke shoulder pain (32, 46). In patients with HSP, there are many reasons for the development of the SASD bursitis such as weak muscle strength, recurrent overuse, posture abnormalities, rotator cuff tendon tears, and muscle imbalance caused by spasms. Currently, as corticosteroids injection into the SASD bursa under ultrasound -guidance (47, 48) provide anti-inflammatory effects by inhibiting the activation of many cytokines, it can decrease inflammatory-dependent pain and is considered as a safe and effective treatment in patients with HSP (13, 24). Additionally, Rah et al. (24) found that subacromial corticosteroid injection in the treatment of HSP, although providing modest short-term benefit, showed improvement in pain reduction and functional improvement. In our study, due to the high incidence prevalence of SASD bursitis in our patient population, it was not surprising that pain relief showed significant improvement in both groups after corticosteroid injection. Therefore, the finding of our study supports current research results showing that ultrasound-guided SASD bursa corticosteroid injection is practical and effective in the treatment of HSP.

However, a prospective cohort study (49) of the efficacy of SASD bursa corticosteroid injection reported good short-term–but poor long-term–outcomes due to the effect of the underlying disease. As is known to all, multifactorial factors such as soft tissue lesions, muscle imbalance, central poststroke pain, and spasticity are responsible for HSP. Few longitudinal studies found that bicipital tendonitis (31, 50) and SASD bursitis (32) are the most common causes, which may also lead to shoulder pain and function limited after stroke. Recent studies showed that LHBT tendinitis and SASD bursitis, which may coexist, were more prevalent in patients with poor upper limb motor function than those with good motor status (32). Given that rotator cuff disorder and LHBT tendinitis may also occur in patients with HSP, it is speculated that SASD bursa injection alone may not be enough to significantly relieve the pain of HSP patients.

LHBT originates at the supraglenoid tubercle and sits between the lesser and greater tuberosities, which is an important source of anterior shoulder pain (51). It has been demonstrated that the upper one-third of the LHBT contains a rich sympathetic innervation network by releasing substance P and calcitonin gene-related peptides which play important roles in neurogenic inflammation (52). Additionally, several histological studies propose that repetitive traction and friction cause the continuous release of proinflammatory cytokines and microscopic degeneration in the LHBT (52, 53). In patients with HSP, LHBT tendinitis may arise due to weak muscle strength, sensory loss posture abnormalities, and uncoordinated muscle movements, which lead to microscopic tears in the tendon, triggering an inflammatory response (22, 32). It's time for us to take effective measures to reverse the progression of degenerative tendinopathy and spontaneous tendon rupture.

In terms of treatment, previous studies found that, due to anti-inflammatory properties, peritendinous corticosteroid injections were widely used in chronic shoulder pain not related to stroke (33, 35, 54). In a randomized comparative study, which investigated the effect of corticosteroid injections in patients with LHBT tendinosis, significant improvements in pain and functional assessment were shown in both groups lasting as long as 6 months after injection (54). In addition, LHBT sheath injection in patients with subacromial compression syndrome has been observed to be effective and fairly safe in reducing pain and improving function (36), which also seems to be an ideal treatment option for HSP. In the course of this treatment, on the one hand, corticosteroid sheath injection could effectively reduce the release of neurogenic inflammatory factors such as substance P and calcitonin gene-related peptide; on the other hand, it could also effectively break the vicious cycle that leads to local tissue damage and inflammation. Moreover, studies have shown that intra-articular corticosteroid injections are reliable and effective in providing pain relief and increasing shoulder PROM in the treatment of HSP (55, 56). It is widely known that the LHBT sheath is connected to the adjacent glenohumeral joint. A recent study showed that ultrasound-guided biceps tendon sheath corticosteroid injections frequently extravasate into the glenohumeral joint (57). Therefore, it is plausible that corticosteroid injection into the LHBT sheath would result in the intraarticular spread and exerts powerful anti-inflammatory effects and pain-relieving properties, reversing the progression of adhesive capsulitis and the LHBT tendinitis. Wang et al. (36) reported that a combined injection of LHBT sheath for patients with shoulder impingement syndrome had a greater effect on pain and function than an injection of SASD bursa alone, consistent with our results. In short, it is not unexpected that both groups experienced pain relief immediately after treatment, but the reduction in pain and increases in some shoulder PROMs (especially those including abduction and external rotation) were significantly higher in the dual-target group compared with the single-target group, Therefore, the present study demonstrated that dual-target injections are more suitable and effective in the pain reduction and functional improvement for patients with HSP than single-target treatments.

Another finding of this study was the significant difference in the shoulder function, PROM, and ADL between groups at each time point, with the dual-target group demonstrating a more significant improvement compared with the single-target participants. These results demonstrated that ultrasound-guided corticosteroid injections improved the motor function and daily living of patients with HSP, in line with several previous reports (24). Moreover, both groups had pain scores that showed a significant negative correlation with FMA and MBI scores. This suggested that along with pain reduction after the injection, both FMA and MBI scores improved at week 4 and week 12 in the follow-up phase. Therefore, we believe that pain reduction and PROM improvement of the affected shoulder might have a positive effect on the activities of daily living and recovery of motor function for HSP patients. In other words, the more obvious the relief of pain, the more patients' limb function and activities of daily living will be improved. Compared with the single-target group, this explained why the upper limb function and daily living activities of the stroke and hemiplegic patients in the dual-target injection group improved more. Sackley et al. (6) reported a negative correlation between MBI score and the high prevalence of shoulder pain after stroke. Our study further supported this finding.

In summary, the complex causes of hemiplegic shoulder pain mean that a single subacromial bursitis injection is insufficient to alleviate the pain. A combination of SASD bursa and LHBT sheath corticosteroid injection is superior to SASD bursa injection alone in reducing shoulder pain and improving functional activities in patients with HSP.

## Limitations

Some limitations of the study should be noted. First, this study had a small sample size and a short follow-up period. As the most important factor in the valuation of treatment is the duration of efficacy, further studies with at least 6 months follow-up are needed to prove the findings and may show greater benefit from injection treatments. Second, there was no placebo group and no only LHBT sheath group in this study. Third, no further ultrasound evaluations of SASD bursa and LHBT regarding structural changes or signal modifications were performed after injection. Fourth, in terms of evaluation of activity of daily living, MBI does not clearly describe the correlation between pain relief and improvement of ADL after stroke because it contains many items that may have nothing to do with the affected shoulder. In addition to pain factors, some factors may have an effect on ADL, including spasticity of the surrounding muscles, limb motor function, and so on. We did not use statistical procedures to reject the above confounding factors, which is also the limitation of this study. Fifth, multifactorial factors may be responsible for HSP (including glenohumeral subluxation, adhesive capsulitis, central poststroke pain, rotator cuff tendon injury, bicipital tendinitis, SASD bursitis, and spasticity of the surrounding muscles), and lead to certain variations of therapeutic effectiveness. Therefore, there was a lack of grouping regarding the cause of HSP.

## Conclusion

Both SASD bursa alone and SASD bursa combined with LHBT corticosteroid injection under ultrasound guidance can improve the clinical symptoms of patients with HSP. Compared with SASD bursa injection alone, the combination of SASD bursa and LHBT injection is more effective and can provide faster pain reduction, increase the PROM of the upper extremity and improve ADL in patients with HSP. Furthermore, the dual-target corticosteroid injection technique can be easily and safely performed under ultrasound guidance and is recommended as a potential alternative option for patients with HSP. More large-scale prospective clinical trials are warranted to further confirm our findings.

## Data Availability Statement

The raw data supporting the conclusions of this article will be made available by the authors, without undue reservation.

## Ethics Statement

The study protocol was approved by Fu Xing Hospital Ethics Committee (Approval Notice Number 2021FXHEC-KY032). The patients/participants provided their written informed consent to participate in this study. Written informed consent was obtained from the individual(s) for the publication of any potentially identifiable images or data included in this article.

## Author Contributions

YW conceived the idea and revised the literature. YH performed the study and wrote the manuscript. TZ, ML, and WL made the clinical evaluation and a substantial contribution to the study. All authors contributed to the article and approved the submitted version.

## Funding

This study was supported by the 2021 Outstanding Talents Project of Xicheng District, Beijing (Fund No. 202131).

## Conflict of Interest

The authors declare that the research was conducted in the absence of any commercial or financial relationships that could be construed as a potential conflict of interest.

## Publisher's Note

All claims expressed in this article are solely those of the authors and do not necessarily represent those of their affiliated organizations, or those of the publisher, the editors and the reviewers. Any product that may be evaluated in this article, or claim that may be made by its manufacturer, is not guaranteed or endorsed by the publisher.
